# Assessment of Sleep in Children with Autism Spectrum Disorder

**DOI:** 10.3390/children4080072

**Published:** 2017-08-08

**Authors:** Makeda Moore, Victoria Evans, Grace Hanvey, Cynthia Johnson

**Affiliations:** Department of Clinical & Health Psychology, University of Florida, 1225 Center Dr., Room 3130, P.O. Box 100165, Gainesville, FL 32610, USA; mmoore0815@ufl.edu (M.M.); torifae@phhp.ufl.edu (V.E.); ghanvey@ufl.edu (G.H.)

**Keywords:** autism spectrum disorder, autism, sleep disturbance, sleep, assessment, questionnaires, actigraphy, polysomnography, videosomnography

## Abstract

Sleep disturbances in children with autism spectrum disorder (ASD) are significantly more prevalent than found in typically developing (TD) children. Given the detrimental impact of poor sleep on cognitive, emotional, and behavioral functioning, it is imperative to screen and assess for sleep disturbances in this population. In this paper, we describe the screening and assessment process, as well as specific measures commonly used for assessing sleep in children with ASD. Advantages and limitations for use in children with ASD are discussed. While subjective measures, such as parent-report questionnaires and sleep diaries, are the most widely used, more objective measures such as actigraphy, polysomnography, and videosomnography provide additional valuable information for both diagnostic purposes and treatment planning. These objective measures, nonetheless, are limited by cost, availability, and feasibility of use with children with ASD. The current review provides an argument for the complementary uses of both subjective and objective measures of sleep specifically for use in children with ASD.

## 1. Introduction

Current estimates suggest that as many as 1 in 68 individuals in the USA are diagnosed with autism spectrum disorder (ASD) and exhibit core symptoms (i.e., deficits in social interaction and communication, and repetitive and restricted behaviors) that often lead to lifelong impairments in various aspects of daily functioning [1,2,3]. Commonly recognized comorbidities associated with ASD include gastrointestinal disorders, epilepsy, sleep disorders, mood disorders, and disruptive behavior [4,5,6,7]. Among these comorbidities, sleep disturbances stand out as the most commonly reported, with prevalence rates up to over 80% for children with ASD [7,8,9,10,11,12,13]. This is in contrast to the estimated 25% of children in the general pediatric population who experience sleep disturbances [14]. In the past, it was believed that sleep disturbances were correlated with level of functioning, but recent research indicates that children with ASD experience higher rates of sleep disturbances across all levels of functioning and cognitive capabilities [15,16,17,18,19].

The sleep disturbances that children with ASD experience most often are described as dyssomnias, which include delayed sleep-onset, poor sleep maintenance/night wakings after sleep onset, and early morning wakings [20,21]. Sleep-onset association problems are especially problematic for children with ASD when the onset of sleep is erroneously associated with specific objects or events (e.g., co-sleeping with a person or pet, wearing particular pajamas, having a particular toy to play with, etc.), such that when absent, sleep cannot be initiated, sleep is delayed to obtain them, or sleep cannot be re-initiated in the event of a night waking [22]. Bedtime resistance is also commonly reported and may relate to a variety of factors including sleep anxiety, sleep-onset association problems, overall non-compliance, over- activity, and general dysregulation [21,23,24,25,26,27].

While understudied, medically based sleep disturbances, such as sleep disordered breathing, parasomnias, movement disorders, and circadian rhythm sleep disorder, most likely occur at a frequency similar to that of the typically developing (TD) pediatric population [28]. Nonetheless, assessment of these types of problems is still warranted given the secondary consequences that are often associated with the problems; for example, sleep disordered breathing (e.g., obstructive sleep apnea), can manifest symptoms similar to Attention-Deficit/Hyperactivity Disorder (ADHD) or other disruptive behavior disorders in TD children.

Parasomnias include disorders of both non-rapid eye movement (NREM) sleep phase and rapid eye movement (REM) sleep phase. NREM sleep disorders include overt behaviors such as sleepwalking, teeth grinding, night terrors, and confusional arousals, and occur during the deeper levels of NREM, typically during the first half of the night [21]. In contrast, REM sleep behavior disorder is characterized by episodes of arousal often associated with vocalizations and/or complex motor behaviors that occur during sleep. Due to the absence of the normal physiologic generalized muscle paralysis, individuals will act out their dreams, which are often very active or violent in content [21]. A couple of reports identify parasomnias as occurring more frequently in children with ASD, but these have relied on parent report, not objective measures [6,29]. For movement disorders in sleep, such as restless legs syndrome, it is not known whether they occur more frequently in children with ASD than in TD children [30]. These diagnoses may be particularly challenging in children with ASD who may not be able to report such movements and who engage in repetitive, stereotypical motor movements separate from sleep disorders. Finally, there is a report that the circadian rhythm responsible for regulation of the sleep-wake cycle could be altered in children with ASD [30]; however, sleep and wakefulness are also influenced by additional biological and environmental factors (e.g., lighting, noise, temperature, etc.).

Many of the aforementioned comorbidities common to children with ASD could negatively impact sleep (i.e., seizure activity, gastrointestinal problems) [7,25]. Additionally, reports in the literature suggest that many of the neurobiological processes that underlie the sleep-wake cycle are altered in children with ASD. Melatonin is a neurohormone that is most known for its role in regulating the sleep-wake cycle [31]. Differences have been observed in the mean plasma melatonin levels and the excretion of melatonin sulfate in children with ASD when compared to the levels observed in TD children in some studies [31,32], but not in others [33]. Treatment outcome studies of supplemental melatonin in children with ASD have also been mixed, but with general agreement that melatonin helps in reducing sleep latency in some children with ASD [34,35,36,37,38]. There is further evidence of alteration in the serotonin systems of children with ASD, and dysregulation of the diurnal cortisol rhythm, which plays a role in regulating circadian rhythm and sleep-wake cycles [21,39].

It was proposed by Johnson [40] that the core social and communication deficits associated with ASD could negatively impact a child’s ability to self-soothe. For example, social-communication deficits may make it more difficult for children to perceive cues from others indicating that it is time to wind down, thus preventing the establishment of bedtime routines. In addition, restricted and repetitive behaviors may delay sleep onset, such as when children do not want to put away preferred toys, engage in specific rituals, perseverate on a particular topic, or have difficulty transitioning from one part of the bedtime routine to another [21,26]. Some studies also suggest that sleep problems may be related to ASD symptom severity. Fewer hours of sleep per night are associated with more ASD characteristics, such as communication problems, stereotypic behavior, and hypersensitivity to the environment [9,11,28,41,42].

The importance of properly assessing children with ASD for sleep disorders cannot be overstated, as inadequate sleep can have detrimental effects on memory, attention, cognition, daytime behavior, and even language acquisition [43,44,45,46,47]. Additional reports suggest that chronic sleep disturbance increases the risk of physical health problems, including changes in cardiovascular, immune, endocrine, nervous system function, and, for children with ASD in particular, increased weight status and poorer overall health-related quality of life [48,49,50,51]. Furthermore, chronic sleep disturbances experienced by children with ASD are less likely to remit with age, and continue even into adulthood [52,53,54]. Therefore, early identification and intervention may help to offset some of the potentially detrimental effects that may occur secondary to prolonged sleep inadequacy.

## 2. Current Assessment Procedures Identifying Sleep Problems

Given the high rates and detrimental consequences of sleep disturbances for children with ASD, it is important to screen for these problems during routine medical care. This initial assessment may include a detailed history of the child’s sleep habits and behaviors that may be associated with sleep or sleep-related activities (e.g., sleep anxiety, bedtime resistance, etc.). Once sleep is determined to be worthy of further assessment, additional steps should be considered. General assessment procedures to identify sleep problems in children with ASD can be categorized by subjective and objective measures. Subjective measures often utilized in the study of sleep in children with ASD generally include parent-report questionnaires or completion of a sleep diary record. In contrast, objective measures of sleep rely less on information from parents, and instead directly measure aspects of sleep through various technologies. Some of the most common objective approaches used to assess sleep in children with ASD include polysomnography, actigraphy, and video-recording. Various studies utilizing these subjective and objective approaches have provided insight into both the strengths and the weaknesses of their utility, which will be discussed in further detail. Table 1 below provides an outline of these measures.

## 3. Subjective Measures of Sleep in Children with ASD

### 3.1. Sleep Questionnaires

Due to social communication deficits and general developmental limitations, most of the information about sleep in children with ASD is gained from parent report. There are numerous sleep questionnaires that have been used to assess sleep in pediatric populations; however, not all of these measures demonstrate adequate psychometric properties [62]. For the purposes of this review, we focus on the measures that are the most widely adopted, and have at least some psychometric validation in children with ASD.

The Children’s Sleep Habit Questionnaire (CSHQ; [55]) is a comprehensive screener of sleep disorders in children. The CSHQ was originally developed to assess sleep problems in TD children aged 4 to 10 years old, based on the pediatric International Classification of Sleep Disorders; however, it has been used to assess sleep disturbance in children of other ages and diagnostic populations, including children with ASD [6,63,64,65,66]. The CSHQ is currently the most widely used standardized sleep assessment tool for children with ASD [10,28,67,68]. The CSHQ produces a total score based on 45 items, and individual subscale scores based on 33 items, with higher scores indicating more severe sleep disturbance. The 33-item scale consists of eight subscales: (1) Bedtime Resistance; (2) Sleep Onset Delay; (3) Sleep Duration; (4) Sleep Anxiety; (5) Night Wakings; (6) Parasomnias; (7) Sleep Disordered Breathing; and (8) Daytime Sleepiness.

Owens and colleagues [55] evaluated the psychometric properties of the CSHQ in a sample of school-aged TD children with a diagnosed sleep disorder and a community sample of healthy TD children, and found that the CSHQ demonstrated adequate internal consistency and acceptable test-retest reliability for both samples. Individual items and subscale scores were also found to accurately discriminate between good and poor sleepers [55], and a total score of 41 as the optimal clinical cut-off for sleep disturbance was determined, using analysis of a Receiver Operator Characteristic curve (ROC). A subsequent study [69] suggested a modified version of the CSHQ for use in children with ASD, given that other studies have been unable to replicate the factor structure of the original 8-subscale version across other samples [70,71,72,73]. Johnson and colleagues [69] found that a modified version with five subscales, constructed via a principle component analysis, demonstrated adequate psychometric properties in the sample of children with ASD; internal consistency for subscales ranged from 0.50 (Sleep Anxiety) to 0.87 (Sleep-onset Association Problems). The new subscales included: (1) Sleep Routine Problems; (2) Insufficient Sleep; (3) Sleep-onset Association Problems; (4) Parasomnias/Sleep-disordered Breathing; and (5) Sleep Anxiety. The authors indicated the relevance of the first three subscales for children with ASD, and also suggested the inclusion of additional items that would be particularly relevant to children with ASD and disruptive behavior, rigidity, ritualistic behaviors and sensory arousal at bedtime, specific sleep-onset association problems, delayed sleep onset, and sleep anxiety [69]. While commonly used, further psychometric development of the CSHQ specifically for use with ASD is suggested.

The Modified Simonds and Parraga Sleep Questionnaire (MSPSQ; [56]) is a screening tool for sleep disturbances in children aged 5 to 18 years old, and has also been used in a modified version as a measure of sleep disturbance and treatment outcome for children with ASD and other developmental delays [23,58,74]. The MSPSQ is composed of 51 items, and is structured in two parts: Part 1 targets sleep quantity and quality, and Part 2 targets specific sleep disorders. The 36 items scored on a Likert scale pertain to six common sleep problem categories: bedtime resistance/struggles, sleep onset delay, parasomnias, sleep disordered breathing, sleep anxiety, and daytime sleepiness [56]. A Composite Sleep Index (CSI) is calculated based on the frequency of problems with settling at bedtime, night waking, early morning waking, co-sleeping, as well as on the duration of settling and night waking. The resulting CSI score ranges from 0 to 12, and a score of 4 or greater indicates a severe sleep problem. The measure includes additional questions to gather information on treatment history for sleep disturbance and the impact of sleep on family functioning. The psychometric properties of this measure have been adequate: Test-retest reliabilities in a sample of children with severe learning disorders ranged from 0.83 to 1.0 [75] with good internal consistency (Cronbach’s *α* = 0.80) and test-retest reliability (Spearman’s rank correlation = 0.83, *p* < 0.01) [76]. In a study comparing the psychometric properties of the MSPSQ and CSHQ in a relatively large sample of children with ASD who were part of the Autism Treatment Network (ATN) but not referred for sleep disturbances, a cutoff total score of 56 on the MSPSQ, determined using a ROC analysis, demonstrated adequate sensitivity and specificity, after identifying poor sleepers using the CSHQ that was completed alongside the MSPSQ upon enrollment [12]. Internal consistency was modest (Cronbach’s α = 0.67). Similar to the CSHQ, the MSPSQ assesses a variety of sleep problem domains, but goes further to obtain information relevant to treatment planning. Additionally, the CSI is brief and has been shown to be sensitive to change.

The Family Inventory of Sleep Habits (FISH; [57]) is another useful measure of sleep that was developed to assess sleep hygiene in children with ASD from ages 3 to 10 years. This instrument gathers information about daytime habits (e.g., “My child gets exercise during the day”), pre-bedtime habits (e.g., “My child sleeps better with certain sheets or blankets on his/her bed”), presence of a bedtime routine (e.g., “My child goes to bed at the same time each night”), sleep environment (e.g., “My child watches TV, videos, or DVDs to help him/her fall asleep”), and parental behaviors surrounding bedtime (e.g., “I stay in my child’s room until he/she falls asleep”). Two versions of the FISH have been developed, including a validated 12-item version [57] that is commonly used in research studies, and a 22-item version that provides a more comprehensive overview of sleep habits [68,77]. Parents rate how often each item has occurred over the most recent month, using a 5-point Likert scale. Total scores range from 12 to 60, with higher scores indicating better sleep hygiene. The 12-item version of the FISH demonstrates modest to adequate psychometric properties: internal consistency (ASD sample Cronbach’s *α* = 0.61; TD sample Cronbach’s *α* = 0.53); test-retest reliability (ASD Spearman’s rank correlation = 0.82, *p* < 0.0001; TD Spearman’s rank correlation = 0.56, *p* < 0.0001) [57]. The FISH may be used to direct intervention planning for children with ASD, as it was developed specifically for this group.

### 3.2. Sleep Diaries

Sleep diaries are another method commonly used to gather information from parents regarding their child’s daily sleep behaviors and pre-bedtime practices. These logs prompt parents to make nightly recordings of specific sleep-related information, including: the time at which the child goes to bed, the time at which the child falls asleep, the time and frequency of night awakenings, the time at which the child wakes in the morning, and the times of daytime naps [67]. Diaries may also include space for parents to report on antecedents, behaviors, and consequences surrounding bedtime. Information collected by sleep diaries aids the identification of sleep problem types and factors that may influence sleep, informs treatment, and provides a means to monitor change over the course of an intervention, as well as an opportunity for parents to receive feedback on their own behaviors [10,36,68,78,79,80]. In comparison to sleep questionnaires, sleep diaries provide the added benefit of collecting information at multiple time points over a period of time, and do not require parents to retrospectively recall information confounded by memory and other preceding factors [58]. The number of diary entries needed to ensure validity remains unclear; however, a minimum of 14 days has been indicated [81,82]. Still, most studies examining sleep in children with ASD only include five days of sleep diary entries [15]. There are limitations to using sleep diaries, in that parents may be unaware of the exact time a child falls asleep or awakens during the night, unless the parent is in the same room or is awakened by the child’s disruptive behavior. Even in this situation, a parent may misinterpret closed eyes or silence as sleep. Sleep diaries may be perceived as laborious by some parents, particularly for parents who are already overwhelmed by the demands of bedtime. Lastly, as with other subjective measures, sleep diaries are vulnerable to reporter bias. Nonetheless, when completed consistently, sleep diaries provide valuable information to help identify sleep patterns and contributing factors, inform treatment, and track treatment progress of sleep disturbances.

## 4. Objective Measures of Sleep in Children with ASD

### 4.1. Actigraphy

An actigraph is a portable, watch-like accelerometer device that detects limb movement, which the actigraph uses as a proxy to measure various parameters of the sleep-wake cycle [59]. Data collected by the watch is then analyzed using a computer software with specialized, age-based algorithms [83]. Software package calculates several sleep variables, such as total sleep time, sleep onset time, morning waking time, frequency of night awakenings, longest sleep period, and sleep efficiency [35]. In regards to how the watch is worn, most studies place the watch on the child’s non-dominant wrist, but alternative placements have been used to improve tolerance (e.g., non-dominant shoulder, ankles, and trunk) [67,84]. Although there is concern for the ability of children with ASD to tolerate actigraph, many studies in this population report adequate compliance and minimal data loss (i.e., less than 20% data loss) [10,23,35,77,85,86] with the exception of one study that lost data from 12 of 20 families, predominantly due to intolerance [68]. Other actigraphy-related issues encountered in this study included device malfunction and inadequate sleep diary entries to prepare the actigraphy data for analysis [68]. With regard to data collection, a minimum of five nights of actigraphy data collection has been generally followed based on Acebo and colleagues’ study [87] where this was determined to be adequate to reliably assess sleep start time, wake minutes, and sleep efficiency in a sample of TD children [88]. However, investigators also noted that certain variables, such as sleep minutes and sleep period, would most optimally be measured over 7 nights or more [87]. One treatment trial successfully collected data over a long period of time, as demonstrated in a 17-week exogenous melatonin treatment trial in children with ASD [35]. Another study found that a structured educational training with parents in actigraphy placement likely contributed to more scorable nights of data for children with ASD, 2–10 years of age [77]. The child wore the actigraph only at bedtime, which was also thought to improve actigraph compliance.

Like other methods, actigraphy use in the assessment of sleep in children with ASD is not without limitations [89]. Actigraphs can be costly, are at risk of being lost, are prone to malfunction, and need to be retrieved for data downloading and data processing. Hence, actigraphy use may be less practical in most clinical settings and in large clinical research trials. At present, there is a lack of standardization across studies with regard to the brand of actigraph, analysis software, and scoring algorithms. The two most commonly used brands in pediatric sleep literature are Ambulatory Monitoring Inc. (AMI; Ardsley, NY, USA) and Philips Respironics Mini-Mitter (PRMM; Philips, Amsterdam, The Netherlands). Data collected by these brands are interpreted equally across studies, despite the differences that exist between the two in sampling mechanism, data processing, and analysis [90]. Furthermore, few studies have compared the validity of these two brands for use in young children, or in children with developmental delays. One study that compared these two brands in TD children aged 3 to 18 years with or without sleep-disordered breathing problems found that, although both devices demonstrated sensitivity in detecting sleep, both showed poor specificity in detecting wakefulness. More importantly, inter-device reliability was found to be poor. Researchers concluded that scoring algorithms should be adjusted depending on age and diagnosis (i.e., healthy versus sleep disordered breathing sample), and that caution should be exercised when comparing results across studies that use different devices [91].

Next, despite actigraphy being an objective measure of sleep, it still relies on parents to appropriately and consistently place the watch on the child before the child goes to bed. Completing this first step may be particularly difficult to implement during activities that are likely challenging (e.g., bedtime), and parents may forget to put the watch on the child at the appropriate time, or forego the device placement due to child non-compliance, among other reasons. Parents may be requested to event mark data [35] (e.g., when lights were turned out, marking events such as the child getting out of bed, taking the watch off). While event marking improves the accuracy of data analysis, it also places an additional demand on parents; therefore, the quality of data marking may vary across participants. To address some of these barriers, Fawkes and colleagues [89] developed guidelines for parents to collect actigraphy data on children with neurodevelopmental disorders. These included defining a clear bed time, showing parents how to event-mark data and what event markings will look like on the graphical output (actogram), assessing parent comprehension through a quiz, completing a sample sleep diary, testing child’s tolerance to the watch through a practice night of recording, and finally providing feedback to parents based on a practice night. Although following such procedural steps do not overcome all potential shortcomings of actigraphy, parent training in use of actigraphy can improve the likelihood of obtaining scorable data.

Lastly, actigraphy relies on limb movement as a proxy of sleep; therefore discrepancies between actigraphy and other sleep measures may be anticipated [23]. For example, sleep onset latency could be underestimated if a child remains awake, but is physically inactive for a significant period of time [58]. Some evidence suggests that actigraphy may be better at detecting sleep compared to wakefulness in clinical populations [84,92]. Still, overall actigraphy data has demonstrated adequate consistency with parent report measures (i.e., sleep diary and questionnaires) for children with ASD [10,77,84], high agreement with polysomnography [58,90,93,94,95,96,97], and has been used in various outcome studies for the treatment of sleep disturbances in children with ASD [23,77,85]. Altogether, actigraphy appears to be a relatively feasible, practical, and widely used method for assessment of specific sleep variables in children with ASD, primarily in research, despite potential tolerance issues. Practical issues limit its widespread use in clinical settings.

### 4.2. Polysomnography

Polysomnography (PSG) is an objective measure of moment-to-moment sleep architecture that is typically performed in a sleep laboratory. Considered the “gold standard” in the assessment of sleep problems [98], PSG involves the recording of many physiological parameters including electroencephalogram, electrooculogram, and electromyogram, as well as electrocardiogram, respiratory measures, and leg muscle activity [60,96]. PSG can identify sleep-related problems that are generally undetectable, or less reliably detectable by other measures, including problems specific to stages of sleep, sleep latency, total sleep duration, and sleep disordered breathing [60,67,99]. In one study, it was shown that children with ASD were more likely to undergo PSG, receive a prescription for continuous positive airway pressure (CPAP), and receive otolaryngol surgical intervention for breathing problems (e.g., adenotonsillectomy, tonsillectomy, and adenoidectomy) compared to children without ASD [100]. 

Conducting PSG, aside from the cost involved, presents other challenges for children with ASD. For PSG to be performed, several electrodes must be attached to the scalp and face throughout the duration of sleep. This may be particularly problematic for children with ASD who are likely to present with sensory differences that make tolerating the sensors difficult [28]. Additionally, PSG is most often performed in a laboratory setting, and the unfamiliarity of such an environment may negatively impact sleep [60]. In line with this concern, a study conducted by Malow and colleagues [28] highlighted key methodological considerations when assessing sleep problems in children with ASD and TD controls. Investigators found that after a single night of laboratory PSG, children with ASD who were poor sleepers experienced significantly more problems with sleep latency and efficiency compared to good sleepers with ASD and TD controls. However, a subsequent night of PSG showed no differences between these groups. Investigators concluded that the PSG abnormalities were more attributable to the child’s ability to adapt to the novel environment, rather than a sleep disorder [28]. Furthermore, they determined that a third night of PSG could address confounds of a novel environment and “catch up” effects (e.g., sleep in excess that children may experience following a night of atypical sleep), presuming that on the third night, the child’s sleep would likely appear more habitual.

More recently, portable PSG equipment has been introduced [101]. Although this method has not yet been widely adopted in pediatric research and practice, portable PSG may provide opportunity for greater ecological validity compared to PSG conducted in a laboratory, based on preliminary outcomes of studies assessing sleep disturbance in samples of adults referred for breathing-related sleep problems [102,103], as well as children with ADHD [104]. Studies examining the feasibility of portable PSG in TD adults with breathing-related sleep problems have found that some participants were unable to easily operate equipment, and found the transportation of equipment to be burdensome [105]. In contrast, other studies found the portable PSG to be quite feasible and well tolerated by young children with and without sleep-related breathing problems [106,107,108]. In particular, the use of a parent-directed systematic desensitization of the portable PSG was shown to improve the success rate of PSG data attainment in the home setting for a sample of children with ASD, developmental delay, and TD controls [108]. Investigators noted that younger children with ASD took significantly longer than other groups to acclimate to the PSG, and experienced higher dropout rates due to intolerability; yet 86% of the subjects with ASD were able to successfully complete the PSG. Overall, studies using PSG have identified decreased time spent in bed, total sleep time, and REM sleep latency, as well as a greater number of muscle twitches in children with ASD compared to healthy controls [17,100,109]. Based on the current findings, PSG appears to be a modestly feasible approach to assessing sleep in children with ASD, and further optimization of this technology can be gained with consideration of desensitizing the child to the devices and utilizing portable PSGs.

### 4.3. Videosomnography

Videosomnography is a portable time-lapse video recording system used to gather objective data on a range of sleep behaviors, including sleep-wake states, frequency and duration of night waking, and other behaviors that one would expect to observe during sleeping periods (e.g., signs of obstructive sleep apnea, limb movements, parasomnias) [61,110]. This also includes parent-child interactions at bedtime that can be used to inform treatment approaches, corroborate subjective reports of sleep and bedtime behaviors, and provide opportunities for parents to receive direct feedback on behaviors observed [23,111]. Unlike PSG, videosomnography cannot detect changes in sleep stages through brainwave activity, oxygen saturation and subtle breathing changes, and eye movements when eyelids are closed [60]. Yet, for children with ASD who may find novel environments aversive, videosomnography can be especially valuable in comparison to PSG, since data is more likely to be collected at the child’s home, and feeling safe in one’s environment has been established as an important aspect of achieving deep sleep in TD individuals [112,113]. Additionally, this method reduces the risk of confounding factors that may otherwise compromise sleep assessment, such as a child’s intolerance to wearing an actigraph, or the intrusiveness of PSG sensors [67,114]. In one of the first videosomnography studies conducted in children with ASD, investigators reported that the method was tolerated by all six children included in the video-recording portion of the study [114]. Furthermore, this approach could offer an alternative approach to the validation of actigraphy, in addition to providing other important idiosyncratic information unattainable by actigraphy alone (e.g., falling asleep specifically on the edge of a particular pillow, waking after sleep onset, etc.) [115].

Videosomnography has several drawbacks. For many young children, cameras and infrared lights can be fascinating, distracting, or even scary (i.e., infrared lights glow bright red in the dark) at bedtime [115]. Children may also sleep underneath bedding for various reasons (e.g., to avoid the camera or seek tactile comfort.), making it virtually impossible to accurately observe behavior [115]. Additionally, among young children with ASD, it is not uncommon for a child to sleep in multiple locations (e.g., falling asleep in living room, being moved to their own bed, waking during the night to move to a parent’s bed). This is problematic when equipment cannot be readily uprooted to the child’s new location [67]. Similarly, this places additional demands on the parents, who are also prone to error in the use of equipment (e.g., forgetting to start recording, knocking down cameras/knocking cameras out of position, etc.) [115]. Overall, videosomnography is used less frequently in sleep research, particularly for children with ASD; however, given the important information it offers that may go undetected by actigraphy or PSG, its tolerability for children with ASD, and its ability to be used as a standalone tool or in combination with other measures, sleep research would benefit from more widespread utilization. Clinically, night video recording, with its ever improving technology, may be valuable in treatment development and planning. Currently, an ongoing study by our group, is utilizing videosomnography, actigraphy, parent questionnaires, and sleep diaries to assess associations between parent-child interactions and sleep.

### 4.4. Other Assessment Considerations

Behavioral functional assessment is another strategy used to examine sleep, and is often employed in conjunction with some of the previously discussed methods, such as videosomnography and sleep diaries. Functional assessment examines the relationship between antecedent and consequence events that may be establishing and maintaining bedtime and other sleep-related problems [116]. This method of assessment has been largely substantiated as a valid approach to various types of problem behaviors [117,118,119], yet this process has been less employed in addressing sleep disturbances [117,120]. This functional method of sleep assessment for children with ASD may yield important information regarding antecedent and consequences maintaining bedtime and sleep behaviors and be useful in developing specific targeted intervention(s) for a particular child [120,121].

Another important consideration in assessing sleep includes obtaining parent-report questionnaires on daytime behavioral functioning. Daytime behaviors, such as hyperactivity and anxiety, may adversely impact the child’s sleep and other behaviors surrounding bedtime directly. Conversely, poor sleep may result in worsening of daytime behaviors. The Child Behavior Checklist (CBCL; [122]) and the Behavioral Assessment System for Children, Third Edition (BASC-3; [123]) are two such measures commonly used in research and clinical practice to assess a variety of internalizing and externalizing behaviors in children. The Aberrant Behavior Checklist (ABC; [124]) is a similar measure that has been commonly used in the assessment of daytime challenging behaviors in children with ASD. The ABC subscales include: Irritability, Social Withdrawal, Stereotypies, Hyperactivity, and Inappropriate Speech [124,125,126,127].

## 5. Conclusions

There is extensive evidence indicating that inadequate sleep can negatively impact cognitive, emotional, and behavioral functioning; therefore, screening and assessing for sleep disturbances in children with ASD is critical to their care [43,44,45,46,47]. While screening may be a brief clinical interview or completion of a questionnaire, diagnoses of a comorbid sleep disorder and intervention planning may require more thorough assessment using a multi-method approach. When choosing assessment methods, it is important to consider the questions to be answered about the sleep disturbance, the feasibility of the methods of sleep assessment, and the burden to be placed on the child and parent/caregivers [62,67]. Subjective assessment approaches have the advantage of cost and time efficiency with minimal burden. However, subjective measures are also generally prone to reporter bias, may not be sufficient for specific sleep disorder diagnoses, and current questionnaires are not specific to the unique presentation of sleep disturbances in children with ASD.

In contrast, objective approaches are useful in providing important diagnostic information, quantifying various sleep parameters, and detecting behaviors that may go unnoticed by parents (e.g., brief night wakings). Still, objective measures rely on the use of devices that may malfunction, are costly, require skilled data download and interpretation, and may be uncomfortable or distracting to children at bedtime. For children with ASD who display sensory difficulties, it would be important to assess tolerance of methods like actigraphy and PSG, as well as the potential impact on sleep even if tolerated, so as to not undermine their utility. For example, even if a child with ASD is able to tolerate PSG sensors, sleep may still be atypical if the child is waking or moving more frequently in order to reduce discomfort caused by the equipment. A study comparing the use of a single question about sleep, parent report on the CSHQ, and objective data from actigraphy showed that relying solely on parent endorsement of whether or not their child has sleep problems may not be enough to accurately screen for sleep disturbances in children with ASD [128]. This can be attributed to the potential for parents to under- or over-report sleep problems, based on their own perceptions of what constitutes a problem.

In the future, development and validation of psychometrically sound instruments for the assessment of sleep and sleep related impairment in children with ASD would improve our ability to assess these domains in a reliable, valid and time efficient manner. The PROMIS (Patient-Reported Outcomes Measurement Information System) [129] consortium funded by the National Institutes of Health have developed two such measures for use in adults [130]. Using these methods toward development and validation, measures could be developed specifically for children with ASD [69]. Further, with ever advancing technology, actigraphs may become increasingly user friendly and less costly. Finally, PSG and videosomnography are likely to improve with streamlined technology and improved ease of use. Using a combination of both objective and subjective measures of sleep disturbance optimizes the potential for accurate diagnosis, therefore improving the ability to develop an appropriate treatment plan to address the sleep problem. Taken together, researchers and clinicians interested in investigating sleep in children with ASD are encouraged to utilize multiple methods to assess sleep disturbance, including both subjective and objective methods, whenever possible to ensure optimal consideration of sleep disturbances and the number of interacting variables that influence them.

## Figures and Tables

**Table 1:** Sleep Measures.

**Subjective Sleep Measures**							
	**Age Range**		**Population Characteristics**		**Items (No.)**		**Subscales/Content**
**The Children’s Sleep Habit Questionnaire (CSHQ) [55]**	4–10 years		Typically-developing ASD (modified)		Total: 45 Subscale: 33		Bedtime Resistance Sleep Onset Delay Sleep Duration Sleep Anxiety Night Wakings Parasomnias Sleep Disordered Breathing Daytime Sleeping
**The Modified Simonds & Parraga Sleep Questionnaire (MSPSQ) [56]**	5–18 years		ASD, other developmental delays (modified)		Total: 51 Likert: 36		Part 1: sleep quantity and quality Part 2: sleep disorders Likert item subscales: Bedtime Resistance/Struggles Sleep Onset Delay Parasomnias Sleep-disordered Breathing Sleep Anxiety Daytime Sleepiness
**The Family Inventory of Sleep Habits (FISH) [57]**	3–10 years		ASD		Total (V1): 12 Total (V2): 22		Daytime habits Pre-bedtime habits Bedtime routine Sleep environment Parental behaviors around bedtime
**Sleep Diaries [58]**	N/A		Typically-developing ASD		N/A		Time at which child goes to bed Time at which child falls asleep Night waking information Morning waking time Daytime nap information Antecedents, behaviors, and consequences
**Objective Sleep Measures**							
		**Setting**		**Procedures**		**Sleep Variables/Disorders**	
**Actigraphy [59]**		Portable		Watch-like device placed on wrist (or leg) to detect limb movement as proxy for sleep; data collected from device using computer software with age-adjusted algorithms		Total sleep time Sleep onset time Morning waking time Frequency of night wakings Longest sleep period Sleep efficiency	
**Polysomnography [60]**		Laboratory Portable		Electrodes placed on scalp and face throughout sleep duration		Sleep latency Total sleep Sleep paralysis Sleep disordered breathing Narcolepsy	
**Videosomnography [61]**		Portable		Time-lapse video recording equipment used to provide visual and auditory data on participant sleeping behaviors		Sleep-wake states Frequency and duration of night wakings Parent-child bedtime interactions	

ASD: Autism Spectrum Disorder; N/A: Not Applicable; V1: Version 1; V2: Version 2.
